# Combinatorial influence of environmental temperature, obesity and cholesterol on SARS-CoV-2 infectivity

**DOI:** 10.1038/s41598-022-08485-6

**Published:** 2022-03-21

**Authors:** Chandi C. Mandal, Mahaveer S. Panwar, Chandra P. Yadav, Vaishnavi Tripathi, Shreetama Bandyopadhayaya

**Affiliations:** 1 Department of Biochemistry, School of Life Sciences, Central University of Rajasthan, NH-8, Bandarsindri, Kishangarh, Ajmer, Rajasthan 305817 India; 2 Department of Statistics, Banaras Hindu University, Varanasi, Uttar Pradesh India

**Keywords:** Computational biology and bioinformatics, Microbiology, Diseases, Risk factors, Mathematics and computing

## Abstract

The continuing evolution of SARS-CoV-2 variants not only causes a long-term global health concerns but also encounters the vaccine/drug effectiveness. The degree of virus infectivity and its clinical outcomes often depend on various biological parameters (e.g., age, genetic factors, diabetes, obesity and other ailments) of an individual along with multiple environmental factors (e.g., air temperature, humidity, seasons). Thus, despite the extensive search for and use of several vaccine/drug candidates, the combinative influence of these various extrinsic and intrinsic risk factors involved in the SARS-CoV-2 virus infectivity has yet to be explored. Previous studies have reported that environment temperature is negatively associated with virus infectivity for SARS-CoV-2. This study elaborates on our previous findings, investigating the link between environmental temperature and other metabolic parameters, such as average total cholesterol and obesity, with the increase in COVID-19 cases. Statistical analysis conducted on a per country basis not only supports the existence of a significant negative correlation between environmental temperature and SARS-CoV-2 infections but also found a strong positive correlation between COVID-19 cases and these metabolic parameters. In addition, a multiphase growth curve model (GCM) was built to predict the contribution of these covariates in SARS-CoV-2 infectivity. These findings, for first time, support the idea that there might be a combinatorial impact of environmental temperature, average total cholesterol, and obesity in the inflation of the SARS-CoV-2 infectivity.

## Introduction

An international public health crisis emanated from Wuhan (Hubei province), China with the emergence of a new coronavirus strain later identified as severe acute respiratory syndrome coronavirus 2 (SARS-CoV-2)^1^. This novel virus spread rapidly across the globe and has been declared as a pandemic by the World Health Organization in March, 2020. As of July, 2021 (data considered in our study), ~ 200 million cases have been confirmed, with over 4 million deaths worldwide. In order to curb the spread of this novel virus, multiple comprehensive measures will have to be devised and thus, it compelled us to elucidate additional factors which might affect the transmission of this virus. Indeed, various nanotechnology based approaches as preventive measures and detection of SARS-CoV-2 have been suggested to combat COVID-19 pandemic^2,3^. Previous studies have demonstrated that environmental temperature affects the spread of other respiratory viruses including rhinovirus, influenza A and B, and other coronaviruses^4^. These data suggested that environmental temperature might also play a role in the transmission of SARS-CoV-2. We recently published an article demonstrating a strong negative correlation between environmental temperature and SARS-CoV-2 infection of a country, worldwide^5^. This conclusion was made at the beginning of COVID-19 pandemic (April, 2020) when the data was too much dynamic and fluid. Thus, to further validate our previous findings, we have analysed the link between monthly average environmental temperature (MAET) and COVID-19 cases of a country by using data from March, 2020 to July, 2021.

Because of the inconsistent results between studies on the role of temperature in SARS-CoV-2 infectivity, we looked into other factors which might also be contributing to the spread of the virus. In this present study, we probed the correlation between average total cholesterol, obesity, and BMI all associated with metabolic processes with confirmed SARS-CoV-2 cases. Apart from being the predominant component of lipid microdomains, cholesterol has also been investigated in the efficient binding of the SARS-CoV virus to the ACE-2 receptor, even before the current COVID-19 pandemic^6,7^. Additionally, a study conducted in Wuhan observed that a number of obesity-associated conditions, including diabetes, hypertension, and coronary artery disease, were common comorbidities in hospitalised patients^8^. Hence, obesity and BMI were the other chosen factors for this study. Statistical analysis identified a strong negative correlation between MAET and the number of COVID-19 cases in a country. Additionally, we identified a positive correlation between the previously mentioned metabolic parameters and the number of COVID-19 cases in the country. This data implicates additional variables, particularly environmental and metabolic factors, collaborates to support the rapid spread of the SARS-CoV-2 infection.

## Results

### Geographical locations of countries with highest levels of average total cholesterol, BMI, obesity, and COVID-19 cases in their population

**Figure 1:**
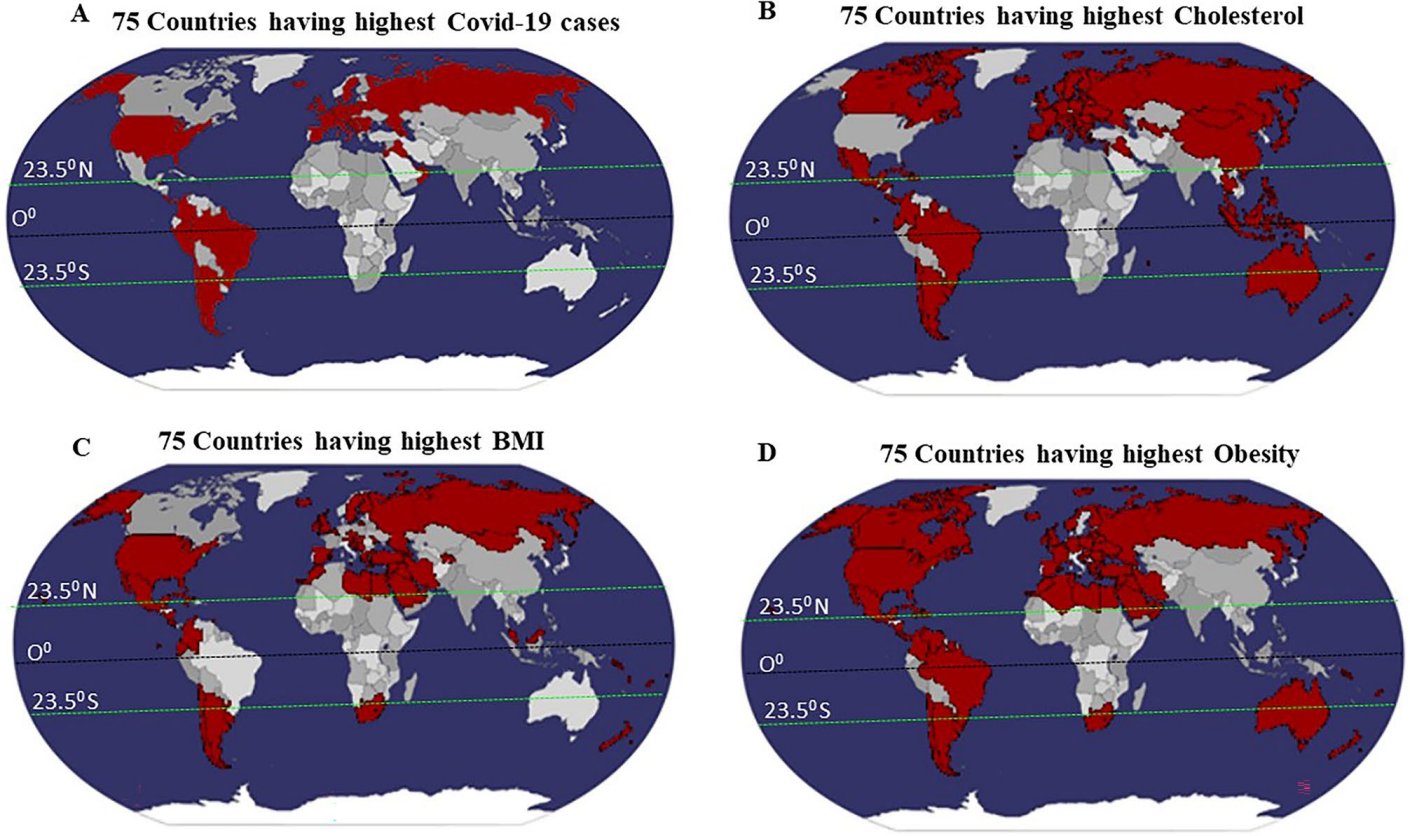
Geographical distribution of the 75 countries with the highest **(A)** total Covid-19 cases per million. **(B)** Average total cholesterol (ATC). **(C)** BMI and **(D)** obesity. Brown color-marked in the map shows the topmost 75 countries with highest total cases per million, ATC, BMI and obesity. Here, Tropic of Cancer (23.5° N), Tropic of Capricorn (23.5° S) and 0° latitudes are shown on a world map. The freely available online server (worldmapmaker.com) was used to make world map.

To investigate whether the geographical location has any impact on SARS-CoV-2 infection, we first identified the top 75 countries for COVID-19 infection (Fig. 1A). As of December 2020, there are 75 countries with at least 14,000 COVID-19 cases, providing a relatively large number for comparative analysis between various parameters. Moreover, 40 out of these 75 countries belong to a relatively colder temperature zone i.e., 15 °C or below (average annual temperature) i.e., above 23.5° N latitude and towards the north pole, which suggests that areas with lower environmental temperature are more prone to SARS-CoV-2 infection. We simultaneously identified the top 75 countries for relatively high average total cholesterol, high-BMI, and prevalence of obesity in their population in the world map in order to associate the influence of these particular metabolic parameters with SARS-CoV-2 infections.

In our recent publication, we investigated whether the novel coronavirus may exhibit increased infectivity in regions with lower environmental temperatures^5^. Additionally, our laboratory had previously reported a negative correlation between environmental temperature and serum cholesterol content of the associated population, based on geographic locations^9^. Furthermore, it was found that the locations with the highest COVID-19 caseloads overlapped with those with the greatest number of metabolic parameters occupying the same locations (Fig. 1B–D). Therefore, these data indicated that these metabolic variables, i.e. high-cholesterol, high-BMI, and high-obesity, might additionally affect viral infectivity.

### Univariate analysis to study the association between COVID-19 cases and metabolic parameters

As stated earlier, our previous publication identified a negative correlation between environmental temperature and the number of novel coronavirus cases^5^. In this earlier publication, the COVID-19 case data was limited to the months of March and April of 2020^5^. Our findings were consistent with other studies^10–12^, detecting significantly lower COVID-19 transmissibility in warmer temperatures. However, some conflicting studies did not find any significant correlation between temperature with COVID-19 cases^13,14^. Generally, such studies were carried out in using only a single country/region. Thus, for this current study, we have expanded our analysis to include data for the total COVID-19 cases per million populations from March, 2020 to July, 2021. Univariate analysis using the Spearman correlation method found a strong negative correlation between MAET and the number of COVID-19 cases in the winter months in comparison to the other months for this period of time, thereby implying that the winter months may promote viral infectivity. A similar trend is also seen when using Pearson correlation analysis. Furthermore, we examined the association between the previously-discussed metabolic parameters and COVID-19 cases. The results of the univariate analysis between these metabolic parameters (average total cholesterol, BMI, and obesity levels) with COVID-19 cases per million are shown in Table 1. In agreement with our previous work, we again found a negative correlation between environmental temperature and COVID-19 cases^5^. Thus, to examine the interdependence of cholesterol levels and total COVID-19 cases, we conducted a univariate analysis between average total cholesterol and the month-wise COVID-19 cases per million by both Pearson and Spearman’s correlation methods. A strong positive correlation was observed between cholesterol and total COVID-19 cases per million of a country (Table 1). Finally, we performed a univariate analysis between COVID-19 case number and BMI of the population of a country and found a positive correlation between these parameters (Table 1). Therefore, the metabolic parameters, i.e., high-cholesterol and obesity, could contribute to novel coronavirus infectivity. However, univariate data analysis alone is not sufficient to address the combinatorial influence of these parameters on virus infectivity.

**Table 1:** Univariate analysis between total COVID-19 cases/million populations of a country and other metabolic parameters.

Months	Pearson	Spearman
**Total COVID-19 cases/million vs. environment temperature**		
March, 2020	−0.4519	−0.5964
April, 2020	−0.3580	−0.4347
May, 2020	−0.0969	−0.2513
June, 2020	0.0183	–0.1450
July, 2020	−0.0030	−0.1424
August, 2020	−0.0213	−0.1978
September, 2020	−0.1030	−0.3119
October, 2020	−0.3553	−0.4934
November, 2020	−0.4875	−0.5496
December, 2020	−0.5141	−0.5530
January, 2021	−0.5096	−0.5741
February, 2021	−0.5273	−0.5870
March, 2021	−0.5440	−0.5848
April, 2021	−0.4470	−0.4768
May, 2021	−0.2878	−0.3582
June, 2021	−0.1231	−0.1998
July, 2021	−0.4519	−0.5964
**Total COVID-19 cases/million vs. BMI**		
March, 2020	0.1010	0.4041
April, 2020	0.1650	0.4762
May, 2020	0.2533	0.4306
June, 2020	0.2907	0.4042
July, 2020	0.3104	0.4015
August, 2020	0.3437	0.4331
September, 2020	0.3681	0.4742
October, 2020	0.3812	0.4940
November, 2020	0.3892	0.4773
December, 2020	0.4143	0.4787
January, 2021	0.4175	0.4836
February, 2021	0.4128	0.4827
March, 2021	0.4159	0.4871
April, 2021	0.4236	0.4883
May, 2021	0.4281	0.4743
June, 2021	0.4269	0.4773
July, 2021	0.4400	0.4789
**Total COVID-19 cases/million vs. average total cholesterol**		
March, 2020	0.2799	0.6805
April, 2020	0.3373	0.6492
May, 2020	0.239	0.5266
June, 2020	0.1702	0.4306
July, 2020	0.1772	0.3891
August, 2020	0.2064	0.3845
September, 2020	0.2498	0.4132
October, 2020	0.3684	0.4847
November, 2020	0.4702	0.549
December, 2020	0.5191	0.5754
January, 2021	0.5372	0.5754
February, 2021	0.5504	0.5784
March, 2021	0.5729	0.5953
April, 2021	0.5829	0.6006
May, 2021	0.5731	0.6043
June, 2021	0.5635	0.6088
July, 2021	0.5623	0.6134
**Total COVID-19 cases/million vs. obesity**		
March, 2020	0.1748	0.5105
April, 2020	0.2918	0.5203
May, 2020	0.352	0.4795
June, 2020	0.3621	0.4642
July, 2020	0.3983	0.4653
August, 2020	0.4208	0.4986
September, 2020	0.4476	0.5384
October, 2020	0.4434	0.5288
November, 2020	0.4498	0.5365
December, 2020	0.4521	0.5371
January, 2021	0.4552	0.5428
February, 2021	0.4566	0.5412
March, 2021	0.4643	0.5454
April, 2021	0.4728	0.5363
May, 2021	0.4542	0.5178
June, 2021	0.4536	0.5274
July, 2021	0.463	0.5259

### Analysis of the distribution of total COVID-19 cases per million from March 2020 to July 2021

**Figure 2:**
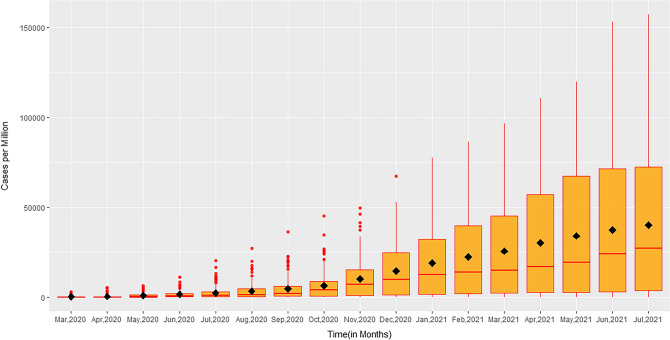
Box plot for total cases per million from March, 2020 to July, 2021.

**Figure 3:**
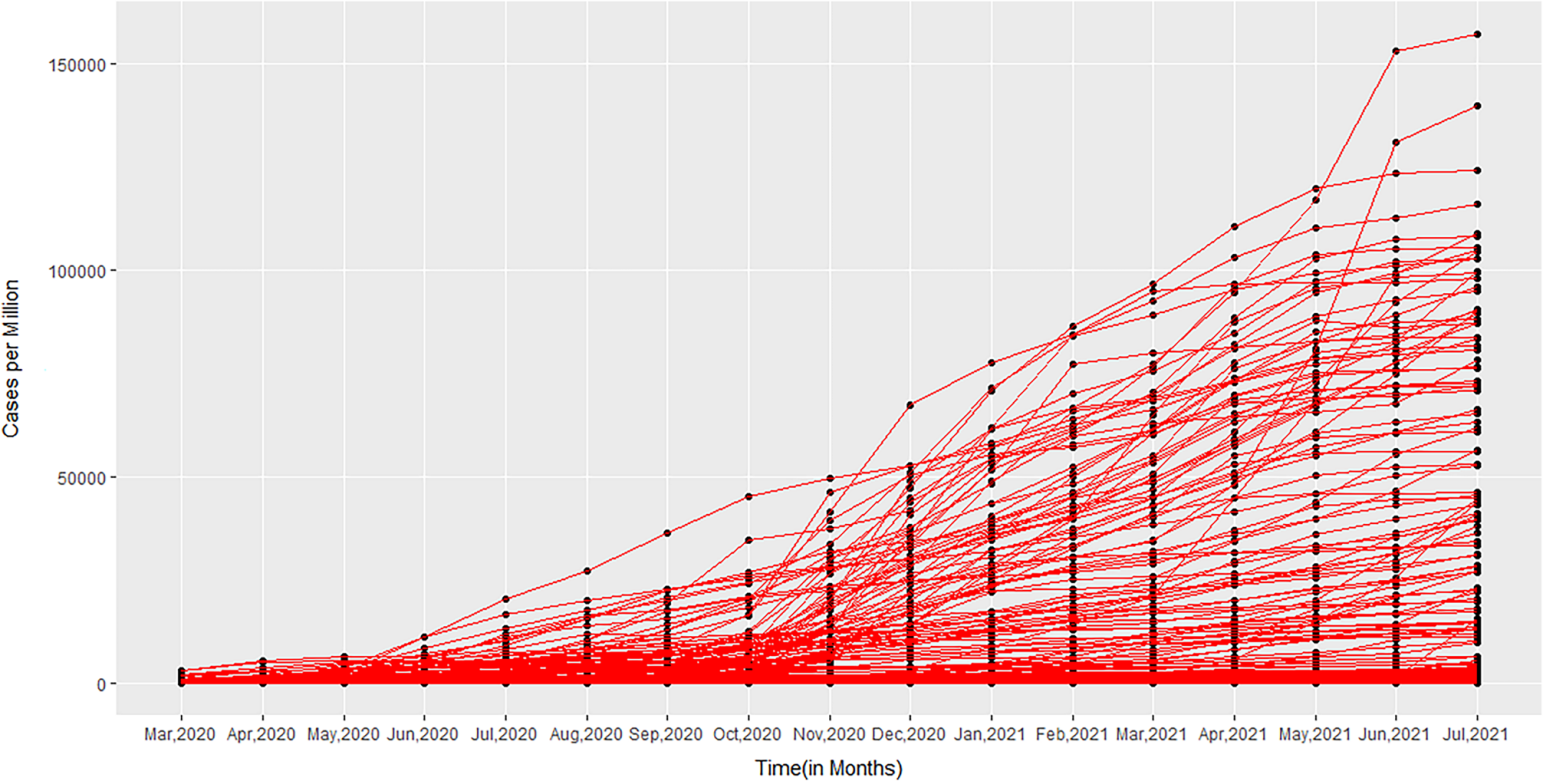
Trajectory plot for total COVID-19 cases per million population of a country from March, 2020 to July, 2021.

To better understand the potential connection between the various factors and the number of COVID-19 infections, further exploratory data analysis was conducted. Figure 2, contains a set of box plots showing the distribution of COVID-19 cases per million over the specified months. These plots show that the mean and median both increase over the period, the mean is larger than the median indicating that the distribution of cases per million is positively skewed for the sample of considered countries. Additionally, the dot points in the initial months indicate that the data points from some countries were outliers. The difference between median and mean increased gradually even in the year 2021, clearly indicating that countries with extreme values were still skewing statistical measures. In addition to this, a simple trajectory plot for the monthly COVID-19 data from March, 2020 to July, 2021 (Fig. 3) showed that in the early months of the year 2020, there is instant growth in COVID-19 cases per million in most of the countries included, in the middle of months of 2020 a stability is seen in all countries and finally, in the last few months, there is again rapid growth in most countries. It can be seen that the rising trend in cases among the countries is non-linear and the growth rate varies from country to country over the defined period.

### Modelling approaches for examining the relationship between environmental temperature, average total cholesterol, and BMI and COVID-19 cases of a country

We used a latent growth curve model (GCM) approach to capture the growth of COVID-19 cases for considered time periods. Furthermore, the multiphase GCM elected to study the intra-individual and inter-individual changes in trajectories for considered countries. In the absence and presence of various covariates, we assigned the ranks to different fitted multiphase models as per their performances. It was observed that model-8 had the lowest AIC and BIC values and large enough TLI value (Table 2), and so it was used for modelling the COVID-19 case number of all countries’ data. The estimates with corresponding standard errors obtained from model-8 are reported in Table 3.

**Table 2:** Multiphase model determining the correlation between different covariates.

Models	Criterion	All countries	Ranking of models
Model 1 (without covariates)	AIC	22,458.97	Rank 8
	BIC	22,589.37	
	TLI	0.6534	
Model 2 (with BMI )	AIC	22,373.56	Rank 4
	BIC	22,506.52	
	TLI	0.7355	
Model 3 (with cholesterol )	AIC	22,385.16	Rank 5
	BIC	22,528.12	
	TLI	0.7189	
Model 4 (with temperature)	AIC	22,348.66	Rank 3
	BIC	22,519.20	
	TLI	0.6853	
Model 5 (with BMI + cholesterol)	AIC	22,392.00	Rank 6
	BIC	22,536.52	
	TLI	0.6966	
Model 6 (with BMI + temperature)	AIC	20,950.00	Rank 2
	BIC	21,068.69	
	TLI	0.6655	
Model 7 (with cholesterol + temperature)	AIC	22,400.65	Rank 7
	BIC	22,582.74	
	TLI	0.7101	
Model 8 (with BMI + cholesterol + temperature)	AIC	20,403.94	Rank 1
	BIC	20,597.59	
	TLI	0.8983	

*AIC* Akaike information criterion, *BIC* Bayesian information criterion, *TLI* Tucker–Lewis Index.

**Table 3:** Estimates for the multiphase model coefficients along with standard error (in small bracket) having time invariant covariates (TICs) and time-varying covariates (TVCs).

Estimates	All countries
**Intercept and slope**	
${g}_{0}$(Var)	65.4469 (−16.1684)
${g}_{1}$(Var)	−0.0004 (−0.0628)
${g}_{2}$(Var)	−1.5834 (497.0114)
${g}_{3}$(Var)	3.9195 (1342.3012)
${\alpha }_{3}$	–
${\alpha }_{4}$	–
${\alpha }_{5}$	–
${\alpha }_{6}$	−1.8668 (1.4132)
${\alpha }_{7}$	−1.9751 (1.3928)
${\alpha }_{8}$	−5.9391(1.4022)
${\alpha }_{9}$	1295.9988 (0.0150)
${\alpha }_{10}$	−7.8613 (1.4035)
${\alpha }_{11}$	0.1068 (0.1601)
${\alpha }_{12}$	–
${\alpha }_{13}$	1.1984 (0.1235)
${\alpha }_{14}$	1.4607 (0.1075)
${\alpha }_{15}$	1.4501 (0.1103)
${\alpha }_{16}$	–
${\alpha }_{17}$	0.7742 (0.0634)
${\alpha }_{18}$	1.0953 (0.0484)
${\alpha }_{19}$	–
**Covariance**	
${g}_{0}$~ ~ ${g}_{1}$	−0.0275
${g}_{0}$~ ~ ${g}_{2}$	30.9401
${g}_{0}$~ ~ ${g}_{3}$	−82.8615
${g}_{1}$~ ~ ${g}_{2}$	0.0088
${g}_{1}$~ ~ ${g}_{3}$	0.1494
${g}_{2}$~ ~ g_3_	−292.1124
**Regression**	
**TICs**	
${g}_{0}$~BMI (${\gamma }_{0}$)	−0.0193 (0.0867)
${g}_{1}$~BMI (${\gamma }_{1}$)	0.0002 (0.0002)
${g}_{2}$~BMI (${\gamma }_{2}$)	−0.3019 (0.1816)
${g}_{3}$~BMI (${\gamma }_{3}$)	0.5621 (0.2715)
${g}_{0}$~CH (${\eta }_{0}$_)_	0.5393 (0.3591)
${g}_{1}$~CH (${\eta }_{1}$_)_	−0.0010 (0.0007)
${g}_{2}$~CH (${\eta }_{2}$_)_	1.0465 (0.7429)
${g}_{3}$~CH (${\eta }_{3}$_)_	−1.1209 (1.1185)
**TVCs**	
C[3] ~ T[3] (${\tau }_{3}$)	−0.0816 (0.0641)
C[4] ~ T[4] (${\tau }_{4}$)	−0.0542 (0.0641)
C[5] ~ T[5] (${\tau }_{5}$)	−0.0226 (0.0640)
C[6] ~ T[6] (${\tau }_{6}$)	−0.0412 (0.0702)
C[7] ~ T[7] (${\tau }_{7}$)	−0.0438 (0.0703)
C[8] ~ T[8] (${\tau }_{8}$)	−0.0588 (0.0731)
C[9] ~ T[9] (${\tau }_{9}$)	−0.0385 (0.1067)
C[10] ~ T[10] (${\tau }_{10}$)	−0.0798 (0.0618)
C[11] ~ T[11] (${\tau }_{11}$)	−0.0402 (0.0634)
C[12] ~ T[12] (${\tau }_{12}$)	−0.0001 (0.0621)
C[13] ~ T[13] (${\tau }_{13}$)	0.0122 (0.0511)
C[14] ~ T[14] (${\tau }_{14}$)	0.0074 (0.0498)
C[15] ~ T[15] (${\tau }_{15}$)	0.0281 (0.0516)
C[16] ~ T[16] (${\tau }_{16}$)	−0.0431 (0.0516)
C[17] ~ T[17] (${\tau }_{17}$)	−0.1959 (0.0531)
C[18] ~ T[18] (${\tau }_{18}$)	−0.2864 (0.0683)
C[19] ~ T[19] (${\tau }_{19}$)	–0.2612 (0.0690)

*TIC* time invariant covariates; *TVC* time varying covariates.

**Figure 4:**
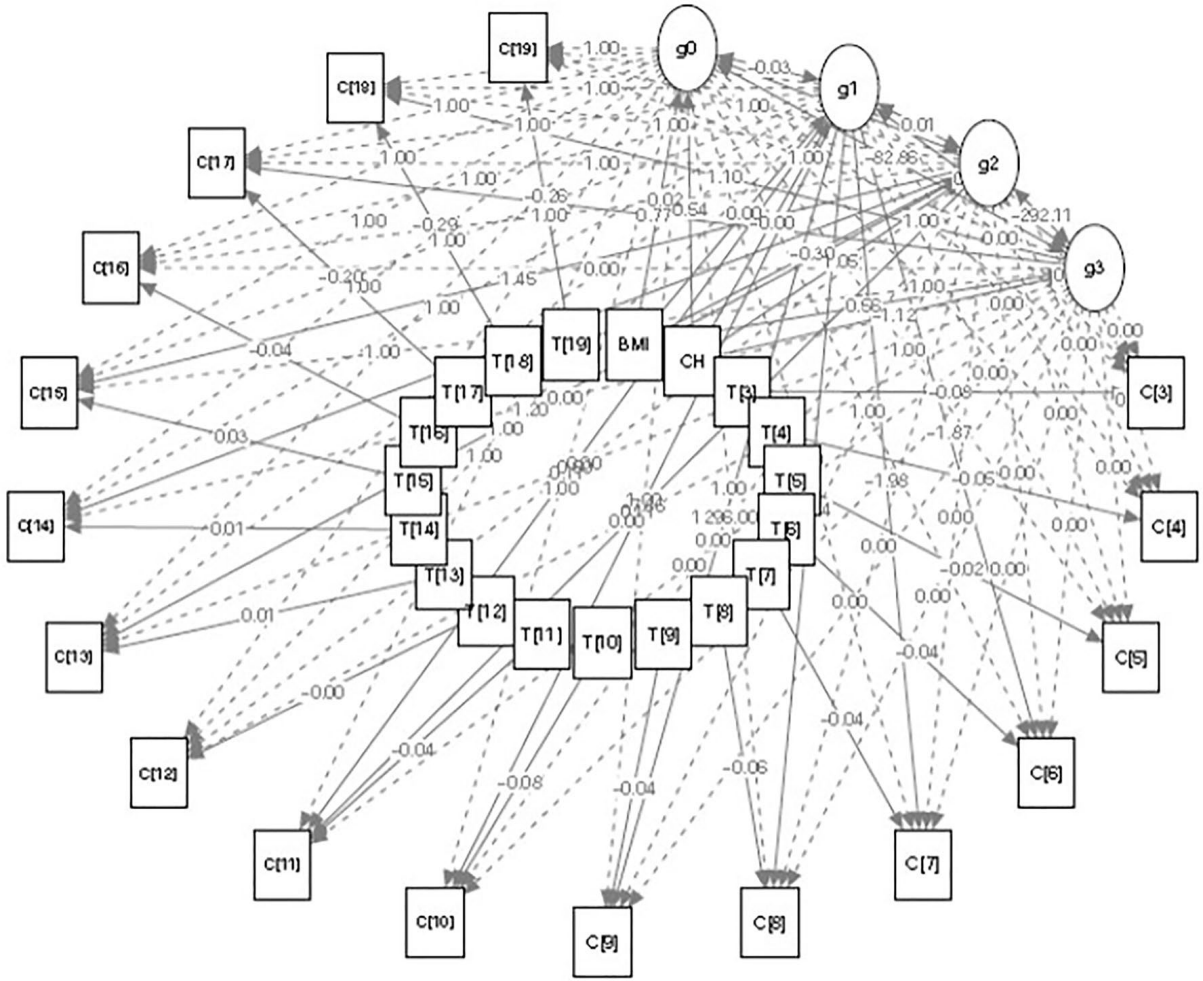
Structure plot for model 8 (multiphase model with temperature + BMI + cholesterol as covariates). Here, *C* represents the outcome variable; *T* represents the monthly average environmental temperature (MAET); *CH *represents cholesterol; ${g}_{0}$, ${g}_{1}$, ${g}_{2}$ and ${g}_{3}$ represents the latent variables. The dotted lines show the fixed coefficients and the bold lines show the estimated coefficients.

From (Table 3), it can be observed that the average baseline value for the outcome variable $C{[t]}_{n}$ is 65.4469 (${g}_{0n}$). The average growth amount in the second, third, and fourth phases (as defined in the “Methods”) are −0.0004 (${g}_{1n}$), −1.5834 (${g}_{2n}$) and 3.9195 (${g}_{3n}$), with significant variances. The estimated parameters of vectors ${A}_{1}\left[t\right]$, ${A}_{2}\left[t\right]$ and ${A}_{3}\left[t\right]$ (${\alpha }_{6}$,${\alpha }_{7},{\alpha }_{8},{\alpha }_{9},{\alpha }_{10},{\alpha }_{11},{\alpha }_{13},{\alpha }_{14},{\alpha }_{15},{\alpha }_{17},{\alpha }_{18})$ can be used to calculate the value of outcome variable $C{[t]}_{n}$ at any month using the estimated values of intercept and slope. The variance terms explain the extent to which countries differ in intra-individual changes, and covariance terms are responsible for the inter-individual differences among countries. Variances differing from zero indicate that these countries differ in their initial levels and slopes of cases per million. For the predictors, it can be inferred that one unit change in BMI is associated with −0.0193 (${\gamma }_{0}$) unit change in slope (${g}_{0n}$), 0.0002 (${\gamma }_{1}$), −0.3019 (${\gamma }_{2}$) and 0.5621 ${\gamma }_{3}$ unit changes in intercepts (${g}_{1n}$, ${g}_{2n}$ and ${g}_{3n}$). Similarly, one unit change in CH is associated with 0.5393 (${\eta }_{0}$_)_ unit change in slope (${g}_{0n}$), −0.0010 (${\eta }_{1}$_)_, 1.0465 (${\eta }_{2}$_)_ and −1.1209 (${\eta }_{3}$_)_ unit changes in slopes (${g}_{1n}$, ${g}_{2n}$ and ${g}_{3n}$). For TVC, one unit change in temperature in March is associated with −0.0816 (${\tau }_{3}$) unit change in $C{[t]}_{n}$. Other coefficients ${\tau }_{4}$, ${\tau }_{5}$, … ,${\tau }_{19}$ can be interpreted similarly. Here, it can be observed that TICs are responsible for the changes in intercepts and slopes. They have an indirect effect on $C{[t]}_{n}$ mediated by latent variables ${g}_{0n}$, ${g}_{1n}$, ${g}_{2n}$ and ${g}_{3n}$, while TVCs have direct impact on outcome variable $C{[t]}_{n}$. A structure plot has also been constructed for the outcome variable, cases per million, alongside the other predictors (Fig. 4). In the structure plot, C[3], C[2], …, C[17] represent the outcome variables while T[3], T[2], …, T[17] represent the MAET. The latent variables ${g}_{1}$, ${g}_{2}$, and ${g}_{3}$ represent the change in the outcome variable in the second, third, and fourth phases respectively. The structure plot depicts the association of temperature with the corresponding month’s outcome variable with coefficients (${\tau }_{t}$). Furthermore, the coefficients of BMI (${\gamma }_{t}$) and cholesterol (${\eta }_{t}$_)_ illustrate an indirect effect on the outcome variable through latent variables ${g}_{0n}$, ${g}_{1n}$, ${g}_{2n}$ and ${g}_{3n}$. The dotted lines in the plot show the fixed parameters, while the bold line corresponds to the estimated coefficients from the model.

## Discussion

The novel coronavirus has spread to pandemic proportions, posing a major threat to the human population with an infection rate that has been found to increase exponentially. This outbreak of COVID-19 has led to millions of deaths worldwide. With the lockdown lifted in many parts of the world, infection rates have been found to increase rapidly. In fact, virus infectivity has rapidly peaked as recently as December 2021. There are multiple environmental and biological factors that could contribute to the rapid spread of disease, such as environmental temperature and metabolic parameters. Studies have reported an association between the infection and transmission of viruses with air temperature and humidity. This has been shown, for example, for the influenza virus ^15^. Reports in the literature suggest that temperature might be an important factor accounting for the transmission of other coronaviruses, like SARS-CoV^16^ and MERS-CoV^17^ because of (i) increased virus half-life at lower temperatures, (ii) greater stability in nasal passages when the epithelial surface is cold, and (iii) greater stability in lower humidity as compared to intermediate humidity^15–17^. Considering these facts, we conducted a study to establish a link between environmental temperature and COVID-19 cases. We observed that countries with more COVID-19 cases were mostly located north of the latitude of Wuhan, China where the pandemic started in December, 2019. Thus, we performed a detailed country-wise statistical analysis which established a significant negative correlation between COVID-19 cases and MAET of a country^5^. However, this initial finding was limited to the COVID-19 data from March and April, 2020. While there are a few studies whose findings are broadly congruous with our own regarding temperature and SARS-CoV-2 caseload^18^, others have found no correlation between temperature and infection rate^13^. In this study, we have validated the relationship between MAET and COVID -19 cases per million from March, 2020 to July, 2021 of a country. Univariate analysis by both Pearson and Spearman’s methods indicated a negative correlation between temperature and COVID-19 cases (Table 1). The statistical analysis also identified a stronger negative correlation for the winter months (November to March) signifying that the warmer months faced fewer SARS-CoV-2 infections compared to the colder months. The box plot (Fig. 2) and the simple trajectory curves (Fig. 3) also demonstrated a high prevalence of COVID-19 infection in the later months of the year as compared to the initial months. To further reconfirm that lower temperatures influence infection rate, we inspected the geographical locations of the countries with moa greater number of infections and observed that most of these countries were located above 23.5^o^N latitude and/or towards the poles, further suggesting that cold temperatures may affect the SARS-CoV-2 transmission (Fig. 1A).

Nevertheless, we did not limit our study exclusively to environmental temperature since there may be multiple reasons for this increase in the novel coronavirus infections. More severe viral infection is observed in those patients who are already suffering from the other pre-existing health complications^19^. Therefore, we refined our research by considering additional metabolic parameters like high-cholesterol, BMI, and obesity, in conjunction with the environmental temperature of a country, in influencing the SARS-CoV-2 caseload. The role of cholesterol in virulence of other respiratory viruses, like influenza, has been well established in several studies. For instance, cholesterol, which maintains membrane structure, is critical for viral stability and virulence^20^. Studies have documented that patients with prior high cholesterol levels are more prone to viral infectivity, eventually leading to severe disease outcomes^21^. Cholesterol-enriched lipid rafts might accommodate the aggregation of ACE2 receptors on the cell membrane, thus enhancing the binding of the S-protein of SARS-CoV-2 to the host cell surface^22^. Another study has shown that individuals with an apolipoprotein (apo) E4 genotype have an increased risk of severe COVID‐19 infection. Though increased cholesterol levels promote ACE2 and furin trafficking inside host cells^21^, cholesterol plasma levels are found to be decreased in patients post-infection. In brief, high cholesterol present in host cell membrane, virus particles, and human blood may augment the virus entry processing in the host cells^21,23^. Our data depicted that geographic locations above 23.5^o^N latitude and towards the poles had a higher prevalence of high average total cholesterol levels, often overlapping the areas with the highest COVID-19 cases (Fig. 1B). Furthermore, the univariate analysis also showed a significant positive correlation between average total cholesterol and COVID-19 total cases per million (Table 1), thereby suggesting that higher cholesterol levels may enhance the infection rate of SARS-CoV-2.

Obesity is a critical health condition which is a consequence of modern sedentary lifestyle. In addition to its other health implications, there is an association between obesity and critical viral infections^24^. Reports have shown that obesity can contribute to the progression of viral infections such as in the case of Hepatitis C infection^25^. Several studies have reported that overweight patients need respiratory support and have increased admission to intensive care units (ICUs) compared to patients with normal weight, even at a younger age^26^. A cohort study showed that obesity is an important factor in disease severity of SARS‐CoV‐2, having the highest impact on patients with a BMI ≥ 35^27^. Moreover, in vitro experiments have shown that ACE2 and TMPRSS2, two essential entry components for SARS-CoV-2 infection, are highly upregulated in the lung epithelial cells of obese patients^28^. Therefore, we explored the relationship between BMI and obesity and COVID-19 caseload. Upon identifying the geographical locations of countries with a higher prevalence of BMI and obesity, significant overlap was observed with those with high numbers of COVID-19 cases (Fig. 1C,D), just as with cholesterol levels. Additionally, statistical analysis identified a positive correlation between the BMI with the total COVID-19 cases per million (Table 1).

Based on these preliminary findings from the univariate analysis data, we conclude that these metabolic parameters i.e., average total cholesterol and BMI, influence the infectivity of the SARS-CoV-2 virus. To authenticate these findings, different statistical approaches were used, and we attempted to model the COVID-19 cases/million trajectory using a latent growth curve model in the presence of time-variant and invariant factors. Multiphase GCM was used to investigate the role of metabolic parameters on the escalation of COVID-19 cases. We evaluated different covariates such as temperature, average total cholesterol, and BMI and tried to fit it in the multiphase models individually with the COVID-19 cases per million and examined the AIC, BIC and TLI values (Table 2). The model having the lowest AIC and BIC values and greater TLC values was considered to be the best fit model fulfilling all the statistical criteria, and based on this, the models were ranked accordingly. Moreover, when these metabolic parameters were evaluated with environmental temperature, it was found to have a greater impact on the infection rate. In order to determine the combinatorial effect of all these factors i.e., environmental temperature, average total cholesterol, and BMI with the COVID-19 cases per million, we incorporated these parameters all together in the multiphase model and determined the AIC, BIC, and TLI values; it was observed that the AIC and BIC values are lowest and TLI is highest for this model in comparison to the other competing models. Therefore, it could be concluded that Model-8 outperformed the other models. The estimates used were obtained from this model. The structure plot for the data including all countries is shown in Fig. 4. Altogether, these findings indicate that patients with higher cholesterol, BMI, and obesity may be more prone to infection, particularly in the winter months. Thus, obese individuals with high average total cholesterol may be at additional risk for getting SARS-CoV-2 infection if they are further exposed to cold environment. This is the first attempt to model COVID-19 cases/million trajectory using the latent growth curve model in presence of time-variant and invariant factors. In fact, such a growth curve modeling approach could be utilized to track and predict the spread SARS-COV-2 infection over the time in presence of the considered factors. This can be helpful to design the policies against the COVID-19.

Although our study indicates a negative correlation between temperature and the number of COVID-19 cases, the ability of this virus to infect might also depend on age, sex, and ethnicity, the prevalence of different diseases in the population, different social distancing practices, and uses of various preventive medicines. Also, our findings are based on the effect of atmospheric temperature on COVID-19 cases; how indoor temperature might affect infection rate is yet to be considered. Our study has considered a rather holistic approach to understanding the role of temperature in infection rates of the virus and takes into account the fluctuations observed in a single country. Moreover, with the emergence of SARS-CoV-2 mutant strains, it is somewhat difficult at this stage to speculate the role of temperature, obesity, and cholesterol on the infection rates of these mutant strains. Although prior SARS-CoV-2 infection protects most individuals from reinfection for at least five months^29^, the first case of COVID-19 reinfection after recovery has been identified in a female from Japan^30^, after which reinfection became a true threat. A recent study has shown that people above 65 years of age have relatively low protection against reinfection by COVID-19^31^. Moreover, recent studies documented that obesity and hyperlipidaemia are associated with lower antibody titre^32,33^. All these findings suggest that various metabolic factors not only enhance the infectivity rate, but also provoke reinfection. However, how metabolic parameters like obesity and cholesterol levels affect the incidence of reinfection still remains unclear. Having said so, the pattern of infection by the virus may differ in the near future due to our growing knowledge of treatment and a much-improved understanding of the SARS-CoV-2 virus infectivity and its associated complications.

Furthermore, this study suggests that individuals with metabolic disorders such as high-cholesterol and obesity could be more susceptible to SARS-CoV-2 infection in the winter months, especially while living in a colder environment. ACE-2 expression in host cells and average total cholesterol levels may be increased in response to exposure to a cold environment and in the winter months^9,34,35^. Elevated ACE-2 levels were observed in multiple metabolic disease conditions such as obesity, diabetes, and higher LDL cholesterol^36^. Additionally, obesity may not only increase ACE-2 and TRMPSS2, but also cellular cholesterol levels by increasing SREBP1^37,38^. Interestingly, a role for low temperature has been suggested in stabilizing the RBD-ACE2 interface and triggering “open” conformations of the COVID-19 spike protein, thus enhancing viral infectivity at cold temperatures^39^. Several studies have also reported multiple roles for cholesterol in enhancing the susceptibility to SARS-CoV-2 infection. Cholesterol-rich microdomains can provide an effective platform for interaction between ACE2 and Spike S-protein^7^. Tang *et. al* reported the role of cholesterol in increasing the density of ACE2 receptors on host cell membranes^23^. Reports using super-resolution imaging have also observed increased SARS-CoV-2 entry in cells with high blood serum cholesterol levels^40^. Furthermore, studies have shown that obesity is a critical factor in COVID-19 severity. Increased ACE-2 expression in lung tissue is seen in patients that are obese, implicating excess adipose tissue in enhancing the spread of the virus^41^. Thus, a colder environment and obesity both increase ACE-2 and host membrane cholesterol, which favour viral entry processing resulting in increase of virus infectivity.

However, other intrinsic factors like hypertension, cardiovascular diseases, renal diseases, and cancer, as well as extrinsic factors like relative humidity and indoor temperature, have not been included in these analyses. Specifically, indoor temperature may contribute to virus infectivity. Actually, cold temperatures and low relative humidity (RH) adversely increase the half-lives of the virus^15^. Aerosolized SARS-CoV-2 has the potential to stay infectious for about 16 h at optimum indoor meteorological conditions^42^. Indoor locations have a relative humidity < 40% which indicates higher chances of airborne SARS-CoV-2 transmission. Moreover, SARS-CoV-2 infection occurs in cool, dry, air-conditioned indoor environments^43,44^ and during cold weather, people mostly stay indoors, which further potentiates transmission^45^. Presently, the USA is setting up policies which suggest maintaining indoor temperatures between 20 and 24 °C and RH around 20–60%^46^. Thus, dry weather generated to maintain indoor temperature (20 °C) in winter months may further potentiate virus infectivity since the virus may persist for longer times in a relatively lower humid atmosphere^47^.

In brief as a conclusive remark, such multiphase growth curve models may be used to depict the contribution of various covariates with COVID-19 cases. Moreover, the individuals with metabolic disorders such as high cholesterol and obesity may have additional risk for this virus infectivity especially in winter months or while living in colder environments. Thus, this study further recommends that a nationwide policy is to be framed in order to combat COVID-19 pandemic and its clinical outcomes for taking care of vulnerable individuals with such metabolic diseases of a cold country. However, further study is required to know whether the infectivity rate of mutant variants of SARS-CoV-2 depends on these factors.

## Methods

### Data collection

COVID-19 case data was collected for every month from March, 2020 to July, 2021 from2021 from ourworldindata.org/coronavirus^48^. We considered the total cases per million as the variable of interest for the COVID-19 case dataset for each month. The environmental temperature data for different countries was collected from *climatestotravel.com*, as described in our recent publication^5^. The latest data for cholesterol and obesity were acquired from the *WHO* website and BMI from *ghdx.healthdata.org*. These datasets are publically available. All data collection was performed in accordance with the relevant guidelines and regulations.

### Geographical distribution of countries

The geographic location of different countries has been marked in the world map with the help of world map marker (worldmapmaker.com), explained previously ^9^. This online server is freely available. The top 75 countries for SARS-CoV-2 infections have been marked in the world map. The top 75 countries for highest average total cholesterol, BMI, and obesity levels in their population have also been marked on the world map.

### Univariate analysis

Univariate analysis was performed using the Spearman and Pearson methods to determine whether to accept the null hypothesis between the two variables, as described previously^5,9,49–51^. Statistical analysis was considered to be significant for $p<0.05$. Univariate analysis was performed using the software GraphPad Prism 6.0.

### Statistical models

For this work, we applied the multiphase latent GCM to study the variable of interest (cases per million) over the defined time period by incorporating time invariant covariates (TICs) and time varying covariates (TVCs). Here, total cases per million over the months was taken as variable of interest while average total cholesterol and BMI were considered as TICs and temperature as TVC. Further, TICs and TVC were examined to see whether the changes in cases per million over months may be explained by the presence of these predictors. R software was used for this analysis. For the modelling purposes, “lavaan” package in the CRAN directory was utilized.

In model fitting, a latent GCM approach was used for COVID-19 cases per million in the presence of different associated covariates: temperature, cholesterol and BMI. The latent GCM captures the growth trajectory of the variable of interest over the defined time period. TICs and TVCs were considered to see whether the changes in cases per million over months may be explained by the presence of these predictors. Here, cholesterol and BMI were treated as TICs, and temperature as TVC. The general structure for the GCM in presence of TIC and TVC can be given as below:

$$C{[t]}_{n}={A}_{0}\left[t\right].{g}_{0n}+{A}_{1}\left[t\right].{g}_{1n}+\cdot \cdot \cdot +{A}_{k}\left[t\right].{g}_{kn}+{\tau }_{t}T{\left[t\right]}_{n}+\epsilon {[t]}_{n},$$

$${g}_{0n}={\vartheta }_{0}+{\gamma }_{0}{BMI}_{n}+{\eta }_{0}{CH}_{n}+{\xi }_{0n},$$

$$\begin{aligned} & g_{1n} = \vartheta_{1} + \gamma_{1} BMI_{n} + \eta_{1} CH_{n} + \xi_{1n}, \\ &\quad \quad \quad \quad ... \\ & g_{kn} = \vartheta_{k} + \gamma_{k} BMI_{n} + \eta_{k} CH_{n} + \xi_{kn} ,\\ \end{aligned}$$

where, $C{[t]}_{n}$ denotes the cases per million for different countries for any particular month t and vectors ${A}_{0}\left[t\right]$, ${A}_{2}\left[t\right]$, …, ${A}_{k}\left[t\right]$ are responsible for capturing the intra-individual trajectories of countries over the months. The latent variables ${g}_{0n}$, ${g}_{1n}$, …, ${g}_{kn}$ represent the slopes and intercepts based on which countries differ inter-individual and ${\tau }_{t}$, ${\gamma }_{k}$, and ${\eta }_{k}$ represent the coefficients associated with the covariates temperature, BMI and cholesterol respectively. Also, ${\vartheta }_{k}$’s are means of slopes and intercepts; ${\xi }_{kn}$’s are residual deviations from latent slope and intercepts with some variances and finally $\epsilon {[t]}_{n}$ denotes time specific residual with variance ${\sigma }_{\epsilon n}^{2}$. The TICs associate directly with the slope and intercepts and have an indirect effect on repeated measures of outcome variables while TVC poses a direct impact on outcome variables. Based on the above equation, several models like linear, quadratic, exponential, and latent GCM have been tested and we found the multiphase GCM to be the most suitable model.

### Multiphase growth curve model

The multiphase GCM is based on two or more regression lines which allows modelling of multiple processes that are responsible for the intra-individual changes over the time periods^52^. As observed from the trajectory plot, the COVID-19 case growth rates were different in the considered months. Therefore, it was more suitable to fit multiple splines than just one line for the defined time slot to capture the COVID-19 cases for different countries. Based on this proposition, we divided the total COVID-19 cases per million over the months into four phases for modelling purposes. Four vectors ${A}_{0}$, ${A}_{1}$, ${A}_{2}$ and ${A}_{3}$ respectively, were used to model the initial or baseline, second, third, and fourth phase. Since, we had the data for seventeen months from March, 2020 to July, 2021, we defined these four phases as Phase I: (March, 2020—May, 2020), Phase II: (June, 2020- December, 2020), Phase III: (January, 2021–April, 2021) and Phase IV: (May, 2021 –July, 2021). The vectors for the chosen multiphase model can be defined as:

$${A}_{0}\left[t\right]= [\mathrm{1,1},\mathrm{1,1},\mathrm{1,1},\mathrm{1,1},1, 1, 1, 1, 1, 1, 1, 1, 1],$$

$${A}_{1}\left[t\right]=\left[0, {0, 0, \alpha }_{6}, {\alpha }_{7}, {\alpha }_{8},{\alpha }_{9},{\alpha }_{10},{\alpha }_{11},1, 1, 1, 1, 1, 1, 1, 1\right],$$

$${A}_{2}\left[t\right]=\left[\mathrm{0,0},\mathrm{0,0}, 0, 0, 0, 0, \mathrm{0,0}, {\alpha }_{13},{\alpha }_{14}, {\alpha }_{15},1, 1, 1, 1\right],$$

$${\mathrm{and}\ A}_{3}\left[t\right]=\left[\mathrm{0,0},\mathrm{0,0}, \mathrm{0,0},\mathrm{0,0},\mathrm{0,0}, 0, 0, 0, {\alpha }_{16},{\alpha }_{17}, {\alpha }_{18},1\right].$$

The parameter ${\alpha }_{i}$’s was estimated from the data. Moreover, the inter-individual differences in the extent to which each process contributes can be governed by the random vectors ${g}_{0}$, ${g}_{1}$, ${g}_{2}$ and ${g}_{3}$. The variances among ${g}_{0}$, ${g}_{1},$ ${g}_{2}$ and ${g}_{3}$ represent the extent to which countries differ on each of the particular aspects of intra-individual change invoked by the corresponding vectors, ${A}_{0}$, ${A}_{1}$, ${A}_{2}$ and ${A}_{3}$ while the covariances among these variables were interpreted as the inter-individual differences in one aspect with respect to another aspect.

Some well-known fitting criteria such as AIC, BIC and TLI were used for choosing the best model. The lower values of AIC and BIC with sufficiently large value of TLI directs to choose a better model. So, a model having lowest values of AIC and BIC with large enough TLI was preferred to others.
